# Validation of survival prediction models for ECMO in Sars-CoV-2-related acute respiratory distress syndrome

**DOI:** 10.1186/s13054-022-04039-4

**Published:** 2022-06-21

**Authors:** Quentin Moyon, Marc Pineton de Chambrun, Guillaume Lebreton, Hédi Chaieb, Alain Combes, Matthieu Schmidt

**Affiliations:** 1 Service de médecine intensive-réanimation, Institut de Cardiologie, APHP Sorbonne Université Hôpital Pitié–Salpêtrière, 75013 Paris, France; 2 Sorbonne Université, INSERM, UMRS_1166-ICAN, Institute of Cardiometabolism and Nutrition, 75013 Paris, France; 3 Service de chirurgie cardiaque, Institut de Cardiologie, APHP Sorbonne Université Hôpital Pitié–Salpêtrière, 75013 Paris, France; 4 Sorbonne Université, Faculté des Sciences Ingénierie, Mathématiques appliquées, Paris, France; 5 Sorbonne Université, GRC 30, RESPIRE, Assistance Publique-Hôpitaux de Paris (APHP) Hôpital Pitié-Salpêtrière, Paris, France; 6 Service de Medecine Intensive Reanimation, iCAN, Institute of Cardiometabolism and Nutrition, Hôpital de la Pitié–Salpêtrière, 47, bd de l’Hôpital, 75651 Paris Cedex 13, France

To the editor

Predictive survival scores have been proposed for patient candidates for ECMO in the context of non-COVID-19 ARDS [1–3]. Indeed, better survival prediction in these patients may improve resource utilization, allow risk-adjusted comparison of centre-specific outcomes, and help clinicians target patients most likely to benefit from ECMO. It could be of utmost importance in the context of a pandemic with a shortage of resources and ICU beds and evolving mortality of the most severe forms with unclear long-term outcomes. However, the performance of these scores in patients with COVID-19 is currently unknown. Based on an ancillary analysis of patients proposed for ECMO consideration at the ECMO–COVID-19 hub in Paris between March 8, 2020, and June 3, 2020 [1], we aimed to validate and compare the performance of the main predictive survival models in that population of COVID-19 patients considered for ECMO.

This study followed TRIPOD recommendations for Prediction Model Development. Ninety-day survival status was prospectively collected for all patients of whom an ECMO was discussed. Briefly, contraindications for ECMO were age > 70 years (case-by-case discussion for those aged between 65 and 70 years), serious comorbidities (including immunosuppression and chronic lung diseases), multiple organ failure, and ongoing mechanical ventilation for > 10 days. Detailed indications and contraindications for ECMO during this period have been listed elsewhere [1]. We computed the Respiratory ECMO Survival Prediction score (RESP) [2], PRedicting dEath for SEvere ARDS on VV-ECMO (PRESERVE) [3], Roch [4], and Sequential Organ Failure Assessment (SOFA) scores (ranging from 3 to 12 pre-ECMO items) in each patient at the time of ECMO consideration. Because the peak pressure was not systematically collected, the plateau pressure was used instead in the RESP score. The discriminative abilities of each score to predict 90-day survival was assessed by the area under the receiver-operating characteristics curves (AUC) and compared to each other using the De Long test. To test whether the observed 90-day survival matched expected mortality in our population, we used the Hosmer–Lemeshow test. Similarly, calibration was tested by the Brier score. The lower the Brier score the more calibrated the prediction.

Among the 575 cases submitted to the ECMO-COVID-19 hub, 302 (56%) patients met eligibility criteria and received ECMO (Fig. 1). The remaining patients were denied ECMO either because of contraindications or because the criteria for ECMO were not met yet. Patients’ characteristics and items of the RESP, PRESERVE, and Roch Scores according to ECMO decision are reported in Table 1. Overall 90-day mortality was 62.6%, whereas it was 54.3% and 73.3% in patients who received ECMO and those denied ECMO (i.e.ECMO contraindications or ECMO criteria not met), respectively.

**Fig. 1:**
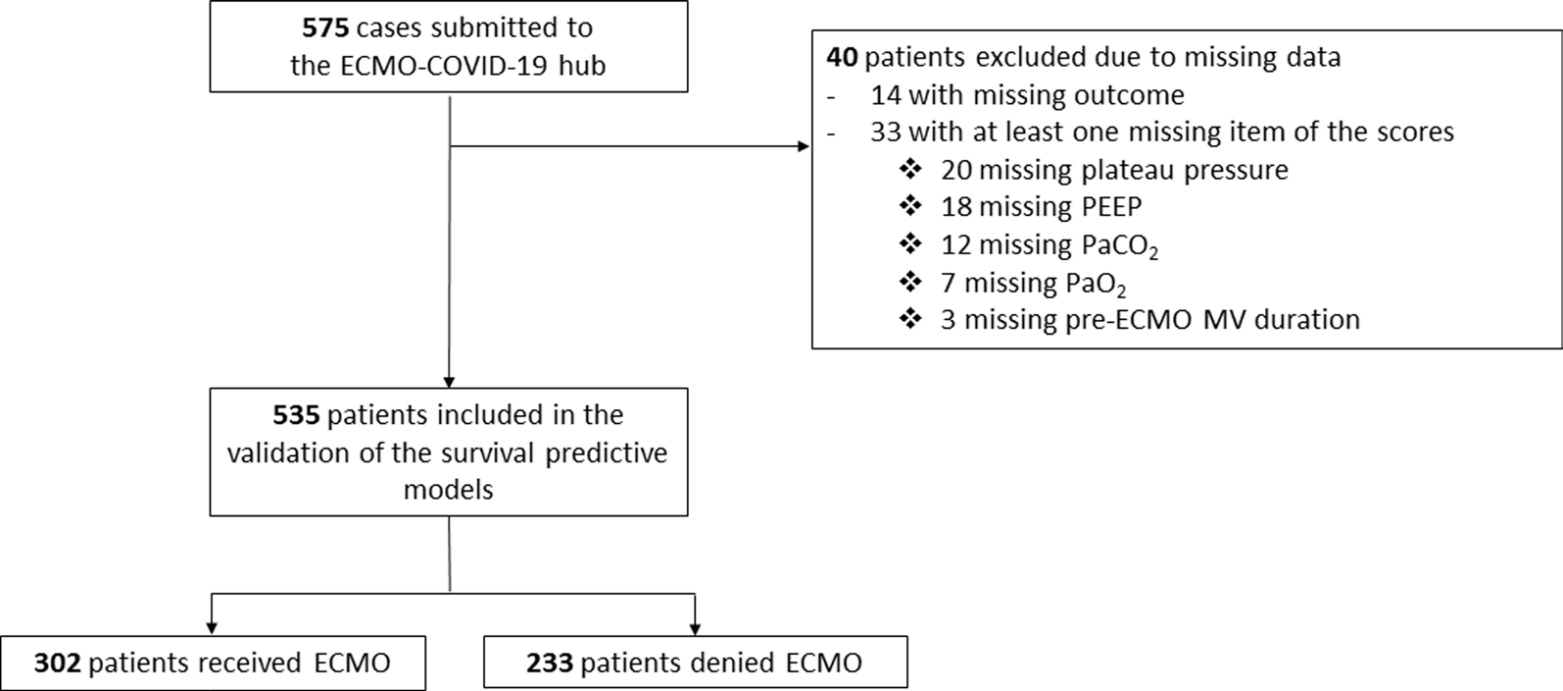
Study flow chart. *ECMO* extracorporeal membrane oxygenation, *MV* mechanical ventilation, *PEEP* positive end-expiratory pressure

**Table 1:** Patients’ characteristics and items of the RESP, PRESERVE, and Roch Scores according to ECMO decision

	Overall, *N* = 535^a^	ECMO decision, *N* = 302^a^	Non ECMO decision, *N* = 233^a^	*p* value^b^
Age (years)	55 (47–61)	52 (44–58)	59 (51–64)	< 0.001
18–49	171 (32)	123 (41)	48 (21)	
50–59	208 (39)	134 (44)	74 (32)	
≥ 60	156 (29)	45 (15)	111 (48)	
Immunocompromised status	42 (8)	18 (6)	24 (10)	0.064
Body mass index > 30 kg/m^2^	260 (50)	143 (48)	117 (52)	0.38
Viral pneumonia	535 (100)	302 (100)	233 (100)	
Central nervous system dysfunction	0 (0)	0 (0)	0 (0)	
Acute associated (non pulmonary) infection	0 (0)	0 (0)	0 (0)	
Cardiac arrest before ECMO	5 (1)	1 (0.3)	4 (2)	0.17
Mechanical ventilation before decision				< 0.001
< 48 h	6 (1)	2 (1)	4 (2)	
48 h–7 days	332 (65)	229 (80)	103 (46)	
> 7 days	172 (34)	56 (20)	116 (52)	
Plateau pressure before decision > 30 cmH_2_O	198 (41)	111 (44)	87 (38)	0.20
PEEP before decision < 10 cmH_2_O	80 (16)	43 (16)	37 (16)	0.84
Prone positioning before decision	498 (95)	284 (94)	214 (96)	0.22
Neuro-muscular blockade agents before decision	512 (96)	291 (96)	221 (95)	0.39
Bicarbonate infusion before decision	0 (0)	0 (0)	0 (0)	–
PaCO_2_ > 75 mmHg	62 (12)	35 (12)	27 (12)	0.88
RESP score	2 (1–4)	3 (2–5)	2 (1–3)	< 0.001
Preserve score	3 (1–4)	2 (0–4)	3 (1–4)	0.029
Roch score	3 (2–4)	3 (3–4)	3 (2–4)	0.89
SOFA score	12 (9–14)	12 (9–14)	12 (9–14)	0.31
> 12	215 (40)	120 (40)	95 (41)	0.81

*ECMO* extracorporeal membrane oxygenation, *RESP* Respiratory ECMO Survival Prediction score, *PRESERVE* PRedicting dEath for SEvere ARDS on VV-ECMO, *PEEP* positive end-expiratory pressure, *SOFA* Sequential Organ Failure

^a^*n* (%); Median (IQR)

^b^Pearson's Chi-squared test; Fisher's exact test; Wilcoxon rank sum test

External validation of the RESP-score in this COVID-19 population demonstrated reasonable discrimination (*c* = 0.74 [95% CI 0.70–0.78]) and good calibration with a Hosmer–Lemeshow C-statistic of 1.56 (*p* = 0.99) in contrast to poorer discrimination of the PRESERVE (*c* = 0.64 [95% CI 0.60–0.70]; *p* < 0.001), Roch (*c* = 0.64 [95% CI 0.60–0.69]; *p* < 0.001), and SOFA scores (*c* = 0.65 [95% CI 0.60–0.69]; *p* = 0.003). Lastly, ninety-day survival was much lower in risk class III and IV (i.e.RESP score ≤  − 2) than in risk class I, II (i.e.RESP score ≥  − 1) (*p* < 0.001) (Fig. 2).

**Fig. 2:**
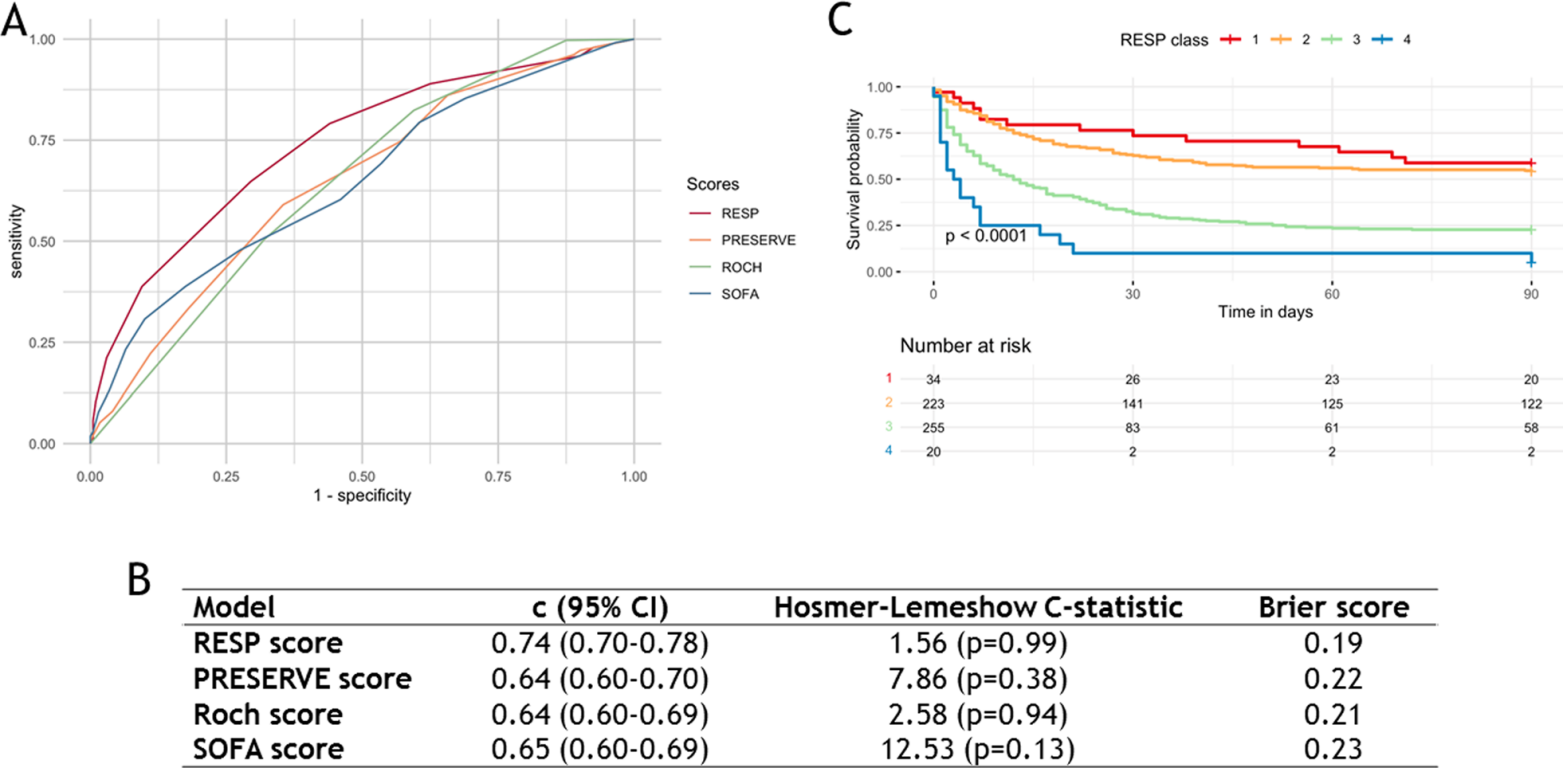
Comparison of **A**) the receiver-operating curves, **B**) Hosmer–Lemeshow C-statistic, and Brier score of the RESP, PRESERVE, Roch, et SOFA scores and **C**) Kaplan–Meier estimates of cumulative probabilities of survival according to the RESP class in a COVID-19 population candidate for ECMO (*n* = 535). *ECMO* extracorporeal membrane oxygenation, *RESP* Respiratory ECMO Survival Prediction score, *PRESERVE* PRedicting dEath for SEvere ARDS on VV-ECMO, *SOFA* Sequential Organ Failure Assessment

To our knowledge, this is the first validation of predictive survival models in a population of patients with severe COVID-19-related ARDS proposed for VV-ECMO. The RESP score exhibited acceptable discrimination and a good calibration which was consistently better than the PRESERVE, Roch, and SOFA scores. Predicting outcomes of COVID patients on ECMO is challenging, as evolving mortality has been reported over the pandemic with changes in the management of the pre-ECMO period and new variants. We confirm the poor discriminant accuracy of the SOFA score to predict mortality of patients with COVID-19, even when combined with age as in the Roch score. The RESP score may offer an additional tool to help clinicians select appropriate COVID-19 candidates for ECMO and improve resource utilization, but it should not be used as a substitute for clinicians’ judgment.

Our study has limitations, the PRESERVE score was initially built to predict 6-month survival [3] and the Roch score was created for patients with influenza-related ARDS [4]. We considered all these cases as “viral pneumonia” in the calculation of the scores although it is likely that some bacterial pulmonary superinfection could have precipitated the need for ECMO. We do not think that it would have changed our results as the distinction between bacterial and viral pneumonia is proposed only in the RESP score and both pneumonia etiologies are finally weighted similarly in that score [2]. Further studies are now warranted to reassess the performance of the RESP score as the pandemic evolves and the expected mortality of patients treated with ECMO is higher. Further adaptation of the RESP score to this specific population could be needed.

## Abbreviations

ARDS: Acute respiratory distress syndrome
AUC: Receiver-operating characteristics curves
COVID-19: Coronavirus disease 2019
ICU: Intensive care unit
RESP: Respiratory ECMO Survival Prediction score
PRESERVE: PRedicting dEath for SEvere ARDS on VV-ECMO
SOFA: Sequential Organ Failure Assessment
VV-ECMO: Venovenous-extracorporeal membrane oxygenation

## Declarations

### Availability of data and materials

The datasets used during the current study are available from the corresponding author on reasonable request.
